# Proteolysis of TAM receptors in autoimmune diseases and cancer: what does it say to us?

**DOI:** 10.1038/s41419-025-07480-9

**Published:** 2025-03-05

**Authors:** Ilona Malikova, Anastassiya Worth, Diana Aliyeva, Madina Khassenova, Marina V. Kriajevska, Eugene Tulchinsky

**Affiliations:** 1 Department of Biomedical Sciences, Nazarbayev University School of Medicine, Astana, 020000 Kazakhstan; 2 Department of Genetics and Genome Biology, University of Leicester, Leicester, UK

**Keywords:** Oncogenes, Bladder cancer

## Abstract

Proteolytic processing of Receptor Tyrosine Kinases (RTKs) leads to the release of ectodomains in the extracellular space. These soluble ectodomains often retain the ligand binding activity and dampen canonical pathways by acting as decoy receptors. On the other hand, shedding the ectodomains may initiate new molecular events and diversification of signalling. Members of the TAM (TYRO3, AXL, MER) family of RTKs undergo proteolytic cleavage, and their soluble forms are present in the extracellular space and biological fluids. TAM receptors are expressed in professional phagocytes, mediating apoptotic cell clearance, and suppressing innate immunity. Enhanced shedding of TAM ectodomains is documented in autoimmune and some inflammatory conditions. Also, soluble TAM receptors are present at high levels in the biological fluids of cancer patients and are associated with poor survival. We outline the biology of TAM receptors and discuss how their proteolytic processing impacts autoimmunity and tumorigenesis. In autoimmune diseases, proteolysis of TAM receptors likely reflects reduced canonical signalling in professional phagocytes. In cancer, TAM receptors are expressed in the immune cells of the tumour microenvironment, where they control pathways facilitating immune evasion. In tumour cells, ectodomain shedding activates non-canonical TAM pathways, leading to epithelial-mesenchymal transition, metastasis, and drug resistance.

## Facts

- Members of the TAM (TYRO3, AXL, MER) family of receptor tyrosine kinases are expressed in professional phagocytes and implicated in efferocytosis and immune suppression.
- TAM receptors are often overexpressed in tumour cells across various cancer types and contribute to drug resistance and metastasis.
- TAM receptors are subjected to proteolysis, resulting in the formation of soluble forms (sTAM).
- sTAM are found in the biological fluids of cancer patients and patients with autoimmune disorders.
- TAM receptors can enter nuclei to generate nuclear pools (nTAM), which activate non-canonical TAM pathways.

## Open questions

- Can sTAM be implemented in clinics as cancer and autoimmunity biomarkers indicating the disease response to TAM-targeted therapy?
- Which cells are a source of sTAM detected in the biological fluids of cancer patients?
- Does TAM ectodomain shedding contribute to the generation of a chronic inflammatory environment that promotes cancer development?
- Are cancer cells more permissive than normal immune cells for generating nTAM and downstream non-canonical signalling pathways?
- What is the role of different nTAM types in various cancer forms in vivo?

## Introduction. Receptor Tyrosine Kinases: proteolytic processing and signalling

There are 58 members of the RTK superfamily in human cells. They mediate cell communications and are implicated in essentially every cellular process, including proliferation, death, migration, and differentiation. As various RTK-induced signalling pathways are aberrantly activated in cancer, the biology of RTKs has attracted immense attention from cancer researchers in past decades [1]. Hundreds of selective inhibitors of RTKs or downstream effectors of RTK-activated pathways are currently in clinical trials as monoagents or parts of combination treatments.

All RTKs represent single-pass transmembrane receptor proteins with extracellular ectodomains (ED), transmembrane domain (TMD), and kinase domain located within the cytoplasmic portions (CD) of the receptor. The abundance and functions of RTKs are controlled at different levels, including proteolytic cleavage and release of ectodomains in the extracellular milieu. Shedding of ED has been attributed predominantly to zinc-containing proteinases termed sheddases that belong to the protein families of MMP (Matrix MetalloProteinases) and the ADAM (Disintegrin And Metalloproteases). ED shedding represents a common approach to regulating RTK functions adopted by cancer cells; it seems that most RTKs are sheddase substrates [2]. The outcome of the shedding depends on various factors. Obviously, removal of the extracellular portion of a receptor blocks ligand-dependent signalling. Moreover, the released extracellular portions of RTKs may retain the ligand-binding capacity and function as decoy molecules to down-regulate signalling. Sequestration of the ligands by shed ED of the receptors was documented for TrkB, c-MET, as well as for TAM family members [3–6].

Sheddases of ADAM and MMP families are regulated at several levels. Tissue inhibitors of matrix metalloproteinases 1-4 (TIMP1-4) are often overexpressed in human cancer tissues and inhibit the activity of the proteinases via direct interactions with their catalytic domains [7]. In addition, co-clustering proteinases and their substrates in lipid rafts and other cell surface microdomains may determine shedding efficacy [8]. Mitogen-activated protein Kinases (MAPK) and Protein Kinase C (PKC) are signalling pathways primarily implicated in modulating the activity of different sheddases. For example, AP-1 and ETS family members, the downstream effectors of MAPK and PKC pathways, are known to cooperate in inducing transcription of MMP-coding genes [9]. The activity of one of the most common sheddases, ADAM17, is also dependent on MAPK pathways. Phosphorylation of the cytoplasmic domain of ADAM17 at T735 by ERK1/2 or p38 leads to the dissociation of ADAM17 from its inhibitor TIMP3 with subsequent induction of the protease activity [10].

In line with these data, the application of lapatinib (a dual inhibitor of EGFR and HER2), BRAF, or MEK/ERK inhibitors attenuated ADAM17-mediated ED shedding and enabled accumulation of active RTKs on the cell surface [5]. Accordingly, cleavage of ERBB4 or VEGFR can be stimulated by cognate ligands or TPA, confirming the involvement of MAPK or PKC in RTK proteolysis [11, 12]. Taken together, these data demonstrate the existence of a negative feedback mechanism which limits the duration of cellular responses to RTKs by their proteolysis. Inactivation of these feedback loops in cancer cells represents a mechanism of acquired resistance to BRAF, MEK or sheddase inhibitors [4, 5, 13].

Several observations make the above model more complicated. In fact, ED shedding may result in activation rather than inhibition of an RTK signalling pathway. Proteolytic cleavage of ERBB2/HER2 results in the formation of an N-terminally truncated membrane-associated protein, p95-HER2, with elevated kinase activity and a transforming potential higher than that of a native receptor [14]. Consistently, p95-HER2 expression was associated with higher aggressiveness and trastuzumab resistance in metastatic breast cancer [15]. The enhanced activity of truncated HER2 protein is reminiscent of constitutively active oncogenic EGFR mutant harbouring deletions within the extracellular domain. This mutant form of EGFR, EGFRvIII detected in glioblastoma multiforme, and other types of cancer exhibits enhanced activity due to inability to bind a ligand resulting in impaired endocytosis and constitutive signalling [16]. Likewise, constitutively active viral homologue vErbB lacks extracellular portion almost completely [17]. More relevant to the topic of this review, a viral homologue of TAM family member MER, v-Eyk, capable of transforming chicken embryo fibroblasts, was discovered in H. Hanafusa’s lab more than three decades ago [18]. This viral oncogene is a constitutively active cytoplasmic tyrosine-kinase lacking transmembrane domain and the entire ED of cellular MER.

ED shedding may result in the degradation of the remaining cytoplasmic portion of an RTK via the ubiquitin-proteasome pathway or the generation of an active membrane-bound fragment with protein kinase activity (as documented for HER2). In addition, cytoplasmic portions of RTKs may undergo Regulated Intramembrane Proteolysis (RIP), a process primed by ED shedding. The release of ED unmasks a second cleavage site that is located within the plasma membrane [19]. This site is targeted by γ-secretase, forming a cytosolic fragment often termed an intracellular domain (ICD). These soluble ICDs possess kinase activity and can be transported to nuclei or mitochondria. RIP is a common phenomenon; at least 27 RTKs have been identified as ICD-producing substrates of γ-secretase [20], and at least two RTKs (IGFR and VEGFR3) are β-secretase substrates [21, 22]. The best-studied RTK in the context of ICD generation is probably the fourth member of the EGFR family, ERBB4, known to promote proliferation of breast cancer cells and tumour growth [23–26].

TAM (TYRO3, AXL, MER) is a family of RTKs implicated in cancer and autoimmune disorders, for which ED shedding and RIP were reported. Recent comprehensive reviews have highlighted the current advances of their targeted inhibition in clinical settings [27, 28]. Here, we outline the biology of these receptors and discuss how TAM-induced signalling pathways are affected by ED shedding in normal and pathological conditions.

## TAM receptors and their ligands: structure, and the role of PtdSer

As with all other RTKs, TAM receptors are single-pass transmembrane proteins. Their extracellular parts consist of two immunoglobulin (IG)-like domains responsible for ligand binding and two fibronectin type III (FNIII) repeats. The intracellular fragments contain tyrosine kinase domains, essential for activating downstream signal transduction pathways [29]. This highly conserved domain contains a KW(I/L)A(I/L)ES motif, which is unique to the TAM family and absent in other RTKs. Activation loops of the kinase domains in TAM receptors contain three tyrosine residues representing autophosphorylation sites. Substituting these residues with phenylalanine individually or in different combinations strongly reduced or abrogated the kinase activity of MER [30]. TAM activation leads to the phosphorylation of distal tyrosine residues, generating docking sites for SH2-containing proteins. Recruitment of GRB2, PLCγ, PI3K p85 and c-SRC has been experimentally shown for activated AXL [31]. Introducing tyrosine-to-phenylalanine mutations into AXL docking sites shows the importance of Y821 (but not that of Y779 or Y866) phosphorylation for crosstalk with c-ABL pathway in head and neck cancer [32]. Likewise, mutating Y867 in mouse MER that is homologous to Y821 in human AXL destroyed docking with GRB2 and abrogated MER function in efferocytosis (see below) [33, 34].

**Fig. 1:**
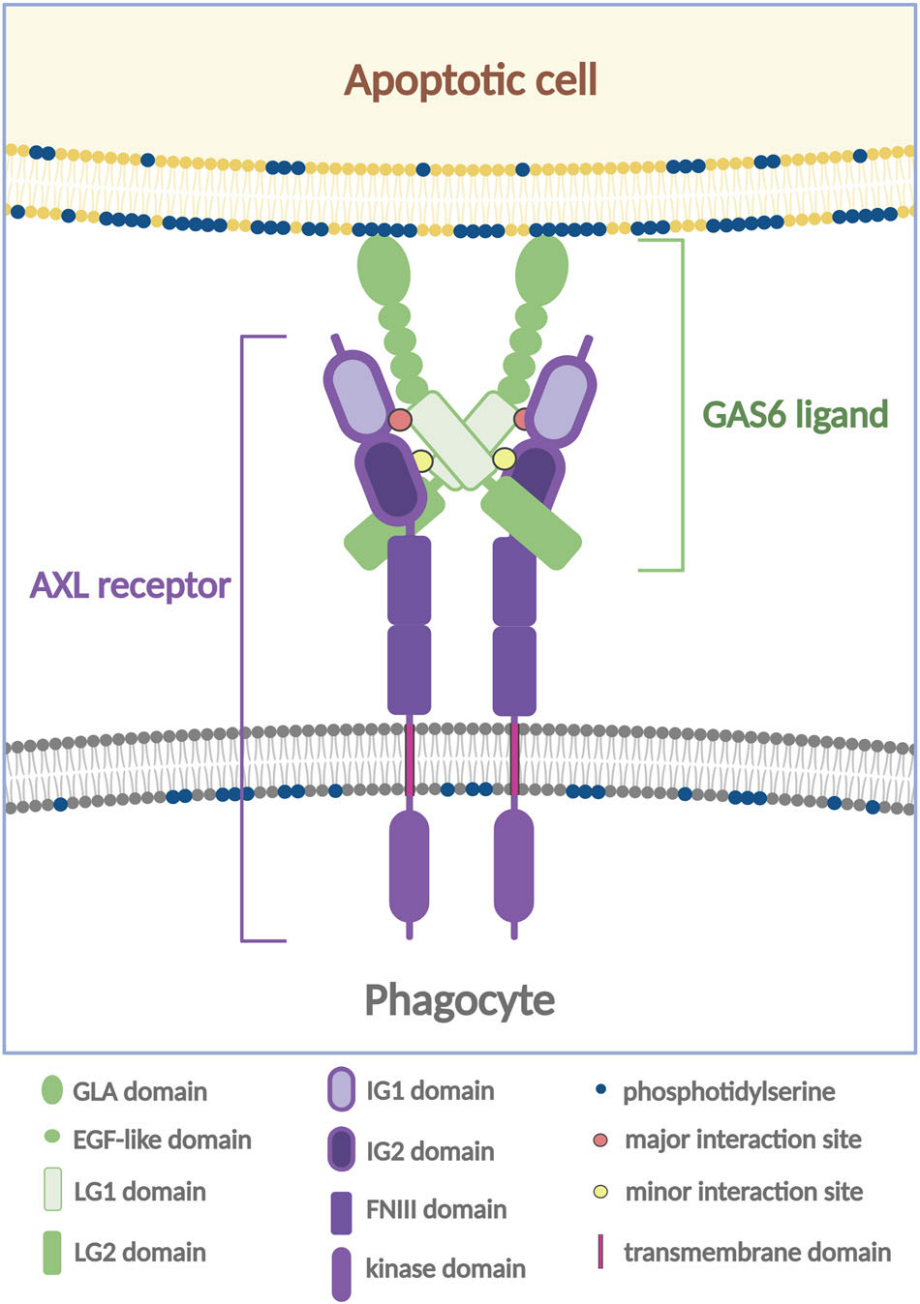
A scheme illustrating a principle of GAS6-AXL interactions. GAS6 LG1 domain contains two sites of interactions with IG1 (strong binding) and IG2 (weak binding) receptor domains. IG1 and IG2 belong to two different receptor molecules [37].

Growth-arrest-specific-6 (GAS6) and Protein S1 (PROS1) are canonical TAM ligands with different affinities for the TAM receptors. GAS6 interacts with all three TAM proteins, preferably with AXL, while PROS1 binds only to TYRO3 and MER. TAM ligands are large proteins with similar structure with the γ-carboxyglutamic acid-rich (GLA) domains at the N-terminus followed by four epidermal growth factor (EGF)-like repeats and sex hormone-binding globulin (SHBG) domains [35, 36]. SHBG domains consist of two laminin G (LG) domains, with LG1 responsible for the receptor binding (Fig. 1). The 3D structure of the complex between AXL and GAS6 defined the binding interfaces between the GAS6 LG1 domain and the pair of AXL IG1 and IG2 domains belonging to the two different molecules [37]. IG1 is responsible for the major interaction site with LG1, while IG2 engages LG1 on a minor site (Fig. 1). The described interaction model is like the mode of PROS1 binding to TYRO3 and MER [38]. Given that the affinity of a ligand to IG2 is low, a high local concentration of the ligand is required for the homodimerization of TAM receptors. The N-terminal GLA domains of TAM ligands undergo vitamin K-dependent γ-carboxylation. This modification allows them to interact with phosphatidylserine (PtdSer) on the external leaflets of some biological membranes, such as plasma membranes of apoptotic cells. As shown for PROS1, cysteine residues of PtdSer-bound TAM ligands may undergo oxidation with subsequent formation of disulphide bonds between adjacent ligand molecules and their oligomerisation [39].

Thus, the locally enhanced concentration of the ligands attached to PtdSer on the membranes of apoptotic cells enables efficient receptor/ligand interactions and activation of intracellular signal transduction. So, the accumulation of GAS6 or PROS1 on the surface of dying cells is a determinant of the function of TAM receptors in efferocytosis, the process of phagocytic clearance of apoptotic cells.

## TAM signalling in efferocytosis

TAM receptors are expressed in a broad spectrum of cells in the adult tissues. Those include endothelial cells, platelets, and, importantly, professional phagocytes, dendritic cells and tissue macrophages, where the function of TAM receptors is well-defined. GAS6 and PROS1 are secreted by TAM-expressing cells or other cells in the same tissue and bind the membranes of apoptotic cells or bodies. TAM ligands exposed on the surface of dead or dying cells ensure their interactions with the phagocytes (so-called “eat-me signals”) and mark them for clearance [40, 41]. TAM-ligand engagement activates PLCγ/PKC, FAK and PI3K pathways. The guanine exchange factor Dedicator Of CytoKinesis 180 (DOCK180) in a complex with scaffold proteins EnguLfment and MOtility 1 or 2 (ELMO1 or ELMO2) are responsible for the spatiotemporal RAC1 activation. This pathway leads to cytoskeletal reorganisation, the building of the so-called phagocytic cup, engulfment and consumption of apoptotic cells [36]. In addition, the TAM/PI3K cell survival pathway ensures the survival of phagocytes, which operate in hostile, toxic environments. The function of TAM receptors in apoptotic clearance is a protective anti-inflammatory measure. It prevents leakage of Danger Associated Molecular Patterns (DAMPs) caused by secondary necrosis, a process that drives autoimmune reactions [42]. In addition to the stimulation of phagocytosis, TAM activation leads to the release of anti-inflammatory cytokines, which suppress the innate immune response [43, 44]. This second arm of TAM signalling involves heterodimerisation of AXL or MER with the type I IFN receptor (IFNAR). The initiated pathway involves GAS6- or PROS1-dependent induction of the Suppressors of Cytokine Signalling 1 and 3 (SOCS1 and SOCS3) and subsequent inhibition of the inflammatory TLR/NF kB signalling [45, 46]. Thus, by activating two signalling modes, TAM receptors control tissue homeostasis by ensuring that apoptosis is a tidy process that does not cause immune responses. So, this surveillance type is important in tissues with high cellular turnover [36].

The importance of TAM receptors in controlling innate immunity was unequivocally demonstrated in mouse KO models. Single *Tyro3*, *Axl* and *Mertk* KO were normal and fertile because of TAM receptors’ functional redundancy. However, double or triple TAM KO animals developed various abnormalities after birth caused by the defects in efferocytosis, prominent accumulation of apoptotic cells and activated lymphocytes in multiple tissues. The abnormalities in TAM KO mice, especially in triple mutants, included various autoimmune conditions resembling systemic lupus erythematosus (SLE), psoriasis, rheumatoid arthritis, nephritis, and multiple sclerosis (MS) in humans [47, 48]. Moreover, mice developed blindness because of the inability of retinal pigment cells to carry out phagocytosis of photoreceptor segments. The impaired phagocytic ability of Sertoli cells in the testes disrupted spermatogenesis and led to male infertility [36, 49].

## TAM receptors in autoimmune diseases in humans

Lessons from experiments in mice have demonstrated that TAM receptors are essential for inhibiting immune responses in different tissues. In agreement with these findings, multiple reports demonstrate an association between TAM deficiency and human autoimmune and inflammatory disorders. Genetic data linking components of TAM pathways with several human autoimmune diseases are consistent with the pathologies observed in mouse models. Namely, polymorphisms in the MER-encoding *MERTK* gene is associated with the susceptibility and the disease course in patients with MS [50, 51], SLE [52], and lupus-associated nephritis in SLE patients [53]. Some of the SNPs were mapped to DNAse I hypersensitivity regions of the gene and associated with reduced *MERTK* expression in monocytes isolated from the blood of MS patients [51, 53]. Likewise, single-nucleotide polymorphisms in GAS6- and PROS1-encoding genes are associated with Behçet’s disease, a condition characterised by multisystemic inflammations with recurrent ocular symptoms and ulcers in different organs [54]. Two SNPs within *GAS6* and *PROS1* genes contributing to the genetic susceptibility of Behçet’s disease were associated with the reduced transcription of corresponding genes.

Several studies have shown diminished TAM signalling in inflammatory disorders due to the decreased expression of the receptors or ligands. Psoriasis is a skin disease triggered by the infiltration and activation of inflammatory cells. The levels of all three TAM proteins and GAS6 in the epidermis of the psoriatic patients were significantly lower than those in healthy skin [55]. Dendritic cells circulating in the blood of MS patients expressed significantly less TYRO3 than healthy controls [56]. In another systemic autoimmune disorder, primary Sjögren’s Syndrome (SS), transcription of *AXL* was significantly reduced in the mononuclear fraction of peripheral blood cells [57]. In several inflammatory and autoimmune diseases, including inflammatory bowel disease, MS, SS, Behcet’s disease, SLE, and psoriasis, plasma levels of GAS6 and PROS1 were lower than in healthy individuals (Table 1).

**Table 1:** sTAM and TAM ligands in the blood of the patients with autoimmune diseases.

Autoimmune conditions				
Disease	Increased sTAM	Ligand	Prognostic importance	References
Behcet’s disease	sAXL	Low GAS6	Correlates with the disease activity	[153]
Granulomatosis with polyangiitis	sTYRO3 sAXL		sAXL discriminated active from inactive disease state.	[154, 155]
Inflammatory bowel disease		Low PROS1		[156]
Multiple sclerosis	sMER sAXL			[116]
Psoriasis		Low GAS6	Predicts cardiometabolic risk	[157]
Rheumatoid arthritis	sMER sTYRO3		sTYRO3 was associated with the total Sharp score, inflammation and disease severity.	[81, 114, 115]
Sjögren’s syndrome	sMER	Low GAS6		[57, 158]
Systemic Lupus Erythematosus	sAXL sMER sTYRO3	Low GAS6 Low PROS1	sMER predicted the disease severity in adult patients. sAxl correlated with the active stage of a juvenile form of the disease.	[109–111, 123, 153, 159]
Systemic sclerosis	sMER		Increased sMER was associated with pulmonary arterial hypertension	[160]

## TAM receptors in cancer

### TAM receptors in TME

Efferocytosis is a common process in the tumour microenvironment (TME) executed by non-professional or professional phagocytes, macrophages, and dendritic cells. Efferocytosis in the TME is pro-tumorigenic; it prevents leakage of immunogenic factors from dead cells. In addition, efferocytosis is associated with releasing inflammation-resolving cytokines, IL4, IL10, IL13, and TGF-β, further facilitating the immune escape of cancer cells [58, 59]. Furthermore, AXL and MER signalling induces expression of PD-L1 and PD-L2 (Programmed death-ligand 1 and 2) immune checkpoint proteins in tumour and myeloid cells inhibiting T-cell anti-tumour activity [60–62]. Macrophages in TME are categorised into M1 and M2 types with tumour-promoting and tumour-suppressive features, respectively. MER signalling shifts the polarisation of macrophages towards the M2 subtype that performs efferocytosis more efficiently than M1 [59, 63]. Pharmacological targeting MER with an ATP-competitive inhibitor of TAM receptors MRX-2843 shifted the balance of macrophage polarisation from M2 to M1 and stimulated anti-tumour immune response in a mouse orthotopic model of glioblastoma [64]. Of note, efferocytosis per se causes transcriptional activation of *MERTK* and cytokine production in the immunosuppressive spectrum [65]. A role for AXL and TYRO3 in efferocytosis performed by macrophages is less clear, but their implication in phagocytic activity in dendritic cells and non-professional phagocytes in TME has been shown [41, 66]. A recent study has demonstrated that in lung TME, the AXL-STAT3 pathway is activated in tumour-associated macrophages, generating a hybrid M1/M2 pool. These cells reside in an aplastic state; they express mesenchymal proteins, tight junction protein ZO-2 and stem cell markers CD44 and CD133. M1/M2 macrophages maintain immunosuppressive TME but also promote angiogenesis [67]. In addition, Natural Killer cells express all three TAM receptors, and their activation by GAS6 or PROS1 suppresses NK cell function and contributes to protumourigenic microenvironment [43]. Various cells in TME, including cancer-associated fibroblasts (CAFs) and tumour-associated neutrophils (TANs), upregulate the expression of TAM ligands, often in response to chemotherapy. GAS6 secreted by TANs and CAFs promotes tumour regrowth and invasion by activating TAM receptors expressed on cancer cells [68–70]. A recent study identified a population of slow-cycling ADAM12+ mesenchymal stromal cells in melanoma, pancreatic and prostate cancer. These cells express GAS6 stimulating AXL-dependent efferocytosis by tumour macrophages, M2 polarisation, immunosuppression and angiogenesis [71].

### TAM receptors in cancer cells

TAM pathways are often hijacked by tumour cells, which may operate as non-professional phagocytes and engulf and ingest their dying neighbours [28, 36]. The ectopic expression of MER in MCF10A and several cancer cell lines stimulated efferocytosis in vitro [72]. In melanoma tissue samples and cell cultures, tumour cells can be engulfed and digested by their neighbours, indicative of efferocytosis, but the implication of TAM receptors was not shown [73]. Nevertheless, TAM receptors are overexpressed in different types of solid and haematological cancers. Their role in cancer has been the research community’s focus during the last decade, and comprehensive reviews on this topic are available [28, 35]. Apoptotic cells are always present in solid tumour masses because of various stresses induced by hypoxia, lack of nutrients, or therapeutic interventions. Therefore, given that TAM signalling activates cell survival pathways (see also “TAM receptors in autoimmune diseases in humans”), the expression of TAM receptors in tumour cells provides a survival advantage.

#### AXL

Among all TAM receptors, AXL is most frequently overactivated in various cancer types [28, 74]. This observation aligns with the reports linking AXL signalling with various pathways activated by stresses or oncogenic mutations. Indeed, transcription of the *AXL* gene is under the control of transcription factors HIF1α, YAP1/TEAD and FRA1/cJUN, which are stress-activated or induced by oncogenic mutations [75–79]. Likewise, mutations in *TP53* may lead to the accumulation of AXL via loss of miR-34, a p53 target microRNA that regulates AXL levels in physiological conditions [80, 81]. In different cancer types, AXL is implicated in epithelial-mesenchymal transition (EMT), a critical determinant of tumour cell plasticity [66]. EMTs are genetic programs operating at different stages of embryonic development controlled by several groups of Zn finger or bHLH transcription factors collectively termed EMT-TFs [82]. During EMT, cells lose epithelial polarity and acquire mesenchymal traits, including invasiveness. Equally important, EMT programs contribute to other hallmarks of cancer, such as cancer cell stemness, immune evasion, and therapy resistance [83, 84]. AXL signalling may directly stimulate EMT in breast, ovarian and pancreatic cancer by activating EMT-TFs SNAIL, SLUG and TWIST; AXL transcription is, in turn, controlled by an EMT-TF ZEB1 [77, 85–87]. Given the cell survival-promoting role of EMT and the fact that the AXL/GAS6 pathway can be stimulated by apoptotic cells in TME, it is not surprising that many reports identified AXL activation as a mechanism bypassing cytotoxic effects of various drugs (reviewed in [66]).

#### TYRO3

Although TYRO3 is often considered an understudied cousin of AXL and MER, important observations are emerging. Triple-negative breast cancers with high TYRO3 expression demonstrated resistance to the immune checkpoint blockade, as was recently shown using syngeneic mouse models. Mechanistically, the TYRO3-driven gene expression program suppressed ferroptosis inflicted by cytotoxic T cells and supported a protumour TME. The authors proposed that death and exposure to the eat-me signals in adjoining cells promoted resistance [88]. In transitional cell carcinoma of the bladder, expression of TYRO3, but not AXL or MER, was strongly elevated compared with normal urothelium. Growth of bladder cancer cells was TYRO3-dependent in vitro and in mouse xenograft models [89, 90]. In CRC, elevated TYRO3 was associated with Duke’s stage and poor patient survival. In vitro, TYRO3 induced expression of the EMT-TF SNAIL and subsequent EMT [91]. EMT activation in CRC cells depended on RIP and the nuclear form of TYRO3 (see next section).

#### MER

MER is most often overexpressed in haematological malignancies, including acute lymphoblastic leukaemia (ALL), acute myeloid leukaemia, and mantle cell lymphoma, among others [28]. As with AXL and TYRO3, MER promotes immune evasion by activating immune checkpoints via PD-L1 and PD-L2 [60]. In addition, MER ensures ALL cell survival under stress; this involves repression of a group of proapoptotic genes, including BAX, PUMA and NOXA, and activation of an anti-apoptotic gene expression signature [92].

Overall, the trio of TAM receptors, significantly contribute to cancer aggressiveness by promoting immune evasion and stress resistance. Equally important, their association with EMT programs triggers tumour cell invasion and metastasis. The underlying mechanisms potentially involve elements of pathways controlling cytoskeletal reorganisation during the formation of phagocytic cups in efferocytosis [66].

## Proteolysis of TAM receptors: sTAM and nTAM

**Fig. 2:**
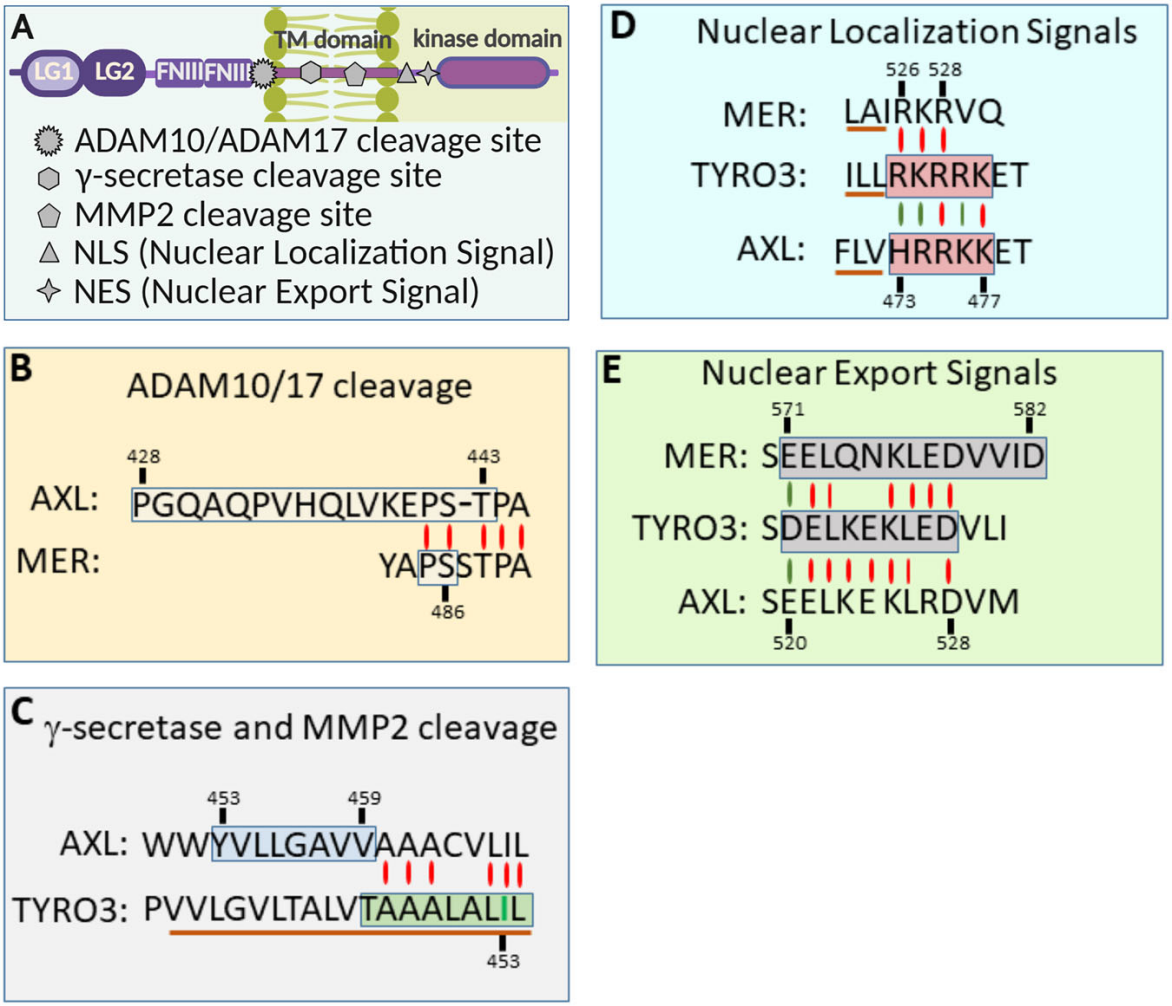
Positions of protease cleavage sites and protein motifs regulating subcellular localisation of TAM proteins. **A** A general scheme illustrating the localisation of the ADAM10/17, MMP2 and γ-secretase cleavage sites, NLS and NES relative to the functional domains of TAM proteins. **B**–**E** The precise mapping of the protease cleavage sites, NLS and NES in TAM receptors [98, 99, 101] Merilahti et al. [20]; Mapped functional sites are indicated with boxes. Parts of transmembrane domains are underlined with the solid brown line. Red and green vertical lines indicate exact matches and similarities, respectively.

ED of all three TAM receptors are detectable in conditioned media of cultured cells and also in biological fluids [20, 93–98]. They are produced by ADAM10 and ADAM17, as shown using specific inhibitors of proteinases and RNA interference. Whereas in MER, the ADAM10/17 cleavage site was identified as Pro^485^-Ser^486^ [95], AXL is cleaved within the fragment Pro^428^-Tre^443^, and ADAM10 is more efficient than ADAM17 in AXL ED shedding [20, 99]. This fragment contains an area of homology with ADAM10/17 cleavage site in MER (Fig. 2A, B). Of note, no homology exists between the amino-acid sequence within the TYRO3 region located N-terminally to the transmembrane domain and other TAM receptors. To our knowledge, proteinases responsible for TYRO3 ED shedding have not been identified to date. Shed domains of TAM receptors act as specific ligand antagonists and dampen TAM-mediated signalling [100].

RIP was reported for all three TAM receptors in NIH 3T3 and several human cancer cell lines. As for other RTKs, ED shedding preceded RIP that was TPA-stimulated and was blocked by the inhibitors of γ-secretase, suggesting a canonical RIP mechanism [20, 99]. Whereas in AXL, the γ-secretase cleavage site was mapped within the Y^453^VLLGAVV^459^ sequence, in TYRO3, a single amino acid substitution (I^449^/A) inhibited the cleavage. This isoleucine residue positions in an area that is non-homologous to the γ-secretase cleavage site in AXL (Fig. 2C). A recent study has shown that in colorectal cancer (CRC) cells, TYRO3 undergoes intermembrane cleavage via a different mechanism involving MMP2 (or gelatinase-A) [98]. Notably, I^449^, critical for the γ-secretase-mediated proteolysis in NIH 3T3 cells, is also a part of the MMP2 cleavage site revealed in HCT116 cells (Fig. 2C).

Several studies have monitored the fate of RIP-generated soluble ICDs of TAM receptors. ICDs of all three TAMs contain nuclear localisation signals (NLS) adjacent to the transmembrane domains (Fig. 2D). Therefore, it is expected that by analogy with ERBB4, ICDs of TAM receptors can be transported to nuclei to form a pool of nuclear TAMs or nTAM. Indeed, nuclear AXL ICD was detected in pancreatic and lung cancer cells treated with the proteasome inhibitor, suggesting that two competing mechanisms, nuclear import and proteasome-dependent degradation, define AXL ICD fate [99]. In addition, nuclear export may represent another important factor: the presence of a nuclear export signal (NES) in MER was predicted in an earlier study [101], and then Hsu and colleagues have shown that the homologous sequence in TYRO3 is a functional NES [98] (Fig. 2E). Consistent with the in vitro data, nuclear localisation of AXL and TYRO3 receptors was reported in cancer tissues, including schwannoma, radioresistant triple-negative breast cancer (AXL), CRC, and leiomyosarcoma (TYRO3) [102–105]. Interestingly, in hepatocellular carcinoma and Jurkat cells, MER nuclear import was controlled by N-glycosylation rather than by RIP [101, 104]. Non-glycosylated nMER was essential for the survival of cells cultured in glucose-deprived conditions [104]. Nuclear forms of AXL and TYRO3 also possess protumourigenic properties. nAXL interacts with the *TP53* gene promoter DNA and represses wild-type *TP53* gene transcription in mesothelioma cells [106]. The association between nAXL and chromatin in the context of mutant *TP53* promoter was not investigated. Interestingly, a full-length nuclear AXL in a complex with WRNIP1 protects metastatic HER2+ breast cancer cells from replication stress-induced apoptosis [107]. In contrast, functional nTYRO3 is RIP-generated; in nuclei, it binds and phosphorylates the acetyl-lysine reader, bromodomain-containing protein 3, BRD3, with the subsequent activation of *SNAIL* gene transcription, leading to the activation of anti-apoptotic and EMT programs [98].

## sTAM and autoimmune diseases

Given an important role for TAM signalling in autoimmune diseases and cancer, their soluble forms in biological fluids received much attention as potential liquid biomarkers for non-invasive diagnosis.

In several autoimmune conditions, plasma levels of sTAM were assessed. A complex multisystem autoimmune/inflammatory disease, SLE, affects both children and adults. The paediatric form is often more severe than the adult-onset disease. In SLE, autoantibodies against nuclear components are detected in the plasma, and it was proposed that impaired phagocytosis leading to secondary necrosis is the underlying cause [108]. In both forms of the disease, adult- and juvenile-onset, the concentration of all three TAMs was significantly elevated in patients’ plasma [109–111] (Table 1). sMER demonstrated a strong correlation with disease activity in adult patients. Moreover, serum from paediatric SLE patients inhibited the phagocytic activity of control macrophages, and the levels of MER receptors on the monocytes in the patient’s blood were reduced. These data suggest that TAM shedding may contribute to the aetiology of SLE [111].

Among autoimmune diseases, SLE is relatively rare, whilst rheumatoid arthritis (RA) is much more common, with a lifetime risk of 3.6% and 1.7% for women and men, respectively [112]. Finding of the new suitable RA biomarkers to improve the accuracy of the diagnosis is an important task [113]. In synovial fluid of rheumatoid arthritis patients, the levels of sTYRO3 and sMER (but not sAXL) were significantly increased [114, 115]. sTYRO3 correlated with the presence of proinflammatory cytokines and inflammation in joints, suggesting that sTYRO3 can be considered a prospective biomarker of RA disease activity.

MS is an autoimmune disease caused by macrophages and microglial cells attacking myelin-containing neuronal fibres. The analysis of chronic active (ongoing demyelination and lymphocyte infiltration) and chronic silent (absence of infiltrating inflammatory cells) MS lesions has shown increased levels of sMER and sAXL in both lesion types. Accordingly, mature ADAM17 levels were high in homogenates from chronic active tissues, and ADAM10 was significantly elevated in chronic active and chronic silent lesions [116].

Enhanced levels of sTAM in biological fluids are not restricted to SLE, RA, MS, and other autoimmune disorders such as Sjogren’s syndrome or Behcet’s disease. Enhanced sTAM levels are detectable in other conditions associated with inflammation and activation of the innate immune system. E.g., higher circulating levels of sMER and more urinary sTYRO3 and sMER were detected in the biological fluids of patients with type 2 Diabetic Nephropathy and liver cirrhosis [117, 118].

The normal physiological function of TAM receptors is in immune suppression. Therefore, the increased levels of circulating sTAM receptors in autoimmune and inflammatory diseases are compatible with the notion that shedding removes functional receptors, generates decoy soluble molecules, and thereby inhibits TAM signalling [72, 94, 119]. Accordingly, both sheddases implicated in TAM processing, ADAM10 and ADAM17, are overactivated in autoimmune diseases [120–123]. Of note, in patients with SLE, ADAM17 expression in peripheral blood mononuclear cells correlated with the levels of sMER in the circulation [123].

In cancer, where TAM pathways generate tumour supporting TME and contribute to cancer aggressiveness, one would expect suppressed shedding and association of sTAM with a better prognosis. Surprisingly, enhanced levels of sTAM are detectable in biological fluids of cancer patients as well.

## sTAM and cancer

The information on sMER and sTYRO3 in the circulation of cancer patients is somewhat incomplete. However, their presence was reported in the plasma of patients with malignant melanoma in association with a poor response to the combined treatment with BRF and MEK inhibitors [5]. In plasma samples of hepatocellular carcinoma (HCC) patients, levels of all three soluble TAM receptors and their ligands were higher than in healthy controls [124]. In addition, sMER and sTYRO3 were detected in conditioned media of various cancer cell lines [20, 94] (Table 2).

**Table 2:** sTAM and TAM ligands in the biological fluids of cancer patients.

Cancer				
Cancer type	Increased sTAM	Ligand	Prognostic importance	Reference
Breast cancer	sAXL (pleural effusion)			[161]
Breast cancer	sAXL (blood serum)		Higher expression in stage IV as compared with stage I cancer	[5]
Hepatocellular carcinoma (HCC)	sAXL		sAXL has a potential as a HCC biomarker	[118, 127, 162]
Hepatocellular carcinoma (HCC)	sAXL	High GAS6		[163]
Hepatocellular carcinoma (HCC)	sTYRO3; sMER; sAXL (plasma)	High GAS6 Low PROS1		[124]
Malignant peripheral nerve sheath tumors (MPNST)	sAXL (plasma)		Predicts neurofibromatosis type 1-related tumor burden	[164]
Malignant melanoma	sAXL; sTYRO3; sMER (plasma)			[5, 126]
Ovarian carcinoma	sAXL (peritoneal effusion)		sAXL concentration increases with the grade	[161]
Pancreatic ductal adenocarcinoma (PDAC)	sAXL (plasma)	High GAS6	sAXL has a potential as a PDAC biomarker	[128]
Renal cell carcinoma	sAXL	High GAS6	Predicts poor survival	[125]

There is more information available regarding sAXL. sAXL is readily detectable in the serum of healthy individuals [119], and its presence and correlation with patients’ survival and disease state were analysed in several cancer types. In renal cell carcinoma, enhanced sAXL levels significantly correlated with poor overall survival of the patients [125]. Likewise, in melanoma patients, sAXL serum levels increased with disease progression and correlated with the shorter survival of patients treated with an immune checkpoint inhibitor Ipilimumab [126]. The analysis of a large cohort of hepatocellular carcinoma (HCC) patients revealed a progressive increase in sAXL levels from healthy controls to low stage and late-stage cancer. Remarkably, sAXL appeared to be a more accurate diagnostic biomarker in HCC than alpha-feta-protein [118, 127]. Similarly, sAXL levels significantly increased in plasma of pancreatic ductal adenocarcinoma (PDAC) patients compared to healthy individuals or patients with chronic pancreatitis (CP). As with HCC, sAXL outperformed the FDA-approved diagnostic biomarker for PDAC, CA19-9, in discriminating PDAC from CP [128] (Table 2).

The question as to which cell types generate sTAM in the blood of cancer patients remains not directly addressed. However, immune cells in the TME are likely not to be the source. Indeed, functional MER receptors expressed in the macrophages of TME are required for tumour growth and metastasis, as shown using mouse models of breast cancer, melanoma, and colon cancer [129]. Functional AXL present in the dendritic cells supported tumour growth via immune checkpoint inhibition [61]. Moreover, TAM receptors are important also for the inhibition of anti-tumour NK activity (see “Proteolysis of TAM receptors: sTAM and nTAM”) [43]. Therefore, it is tempting to speculate that sTAM are generated by malignant cells in tumour tissues rather than by the immune cells in TME. This is in accord with the reported presence of sTAM in the conditioned media of tumour cells in the culture.

**Fig. 3:**
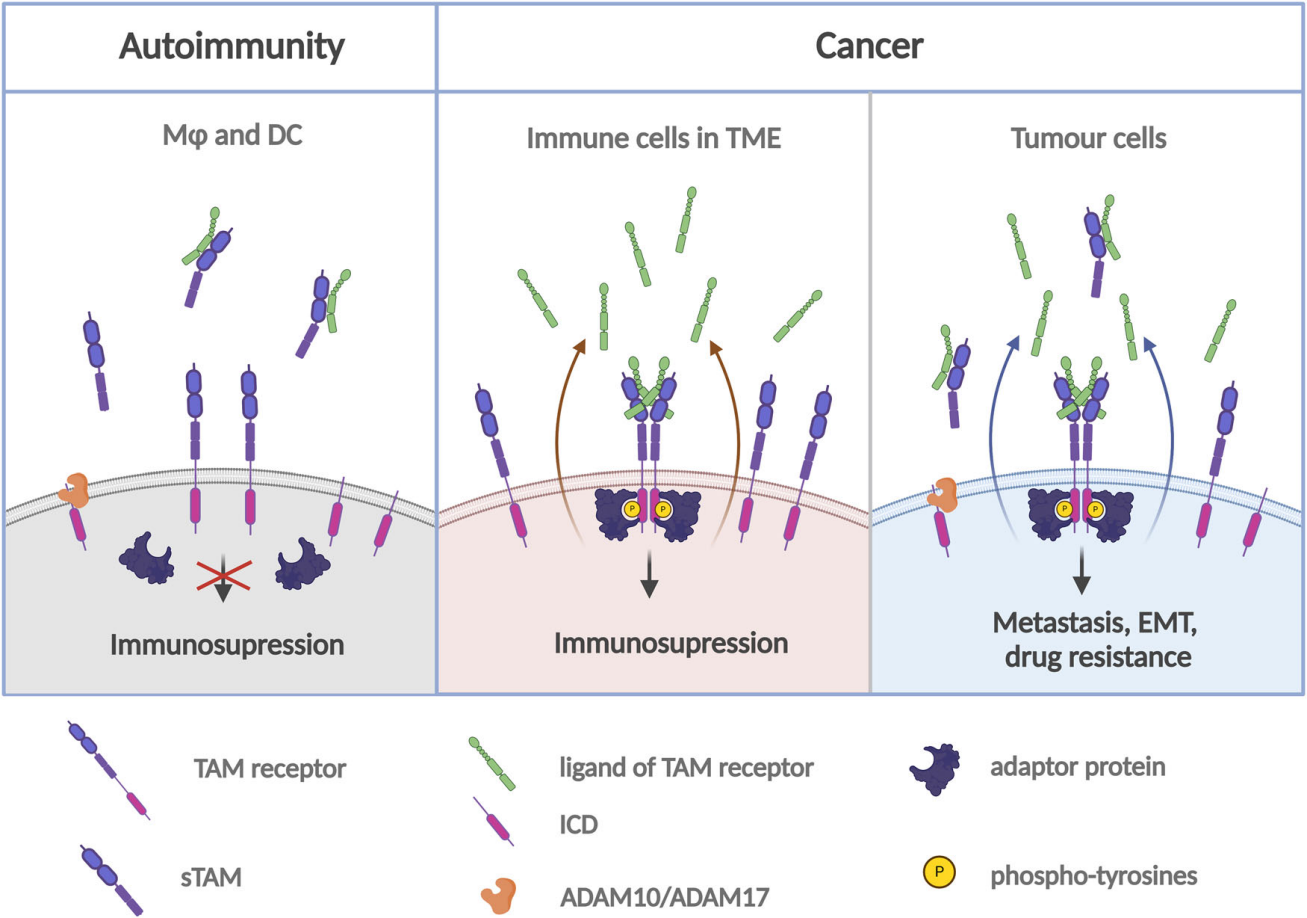
Impact of TAM receptor ectodomain shedding on cell signalling in autoimmune diseases and cancer. In autoimmune diseases, the shedding of TAM receptors inhibits canonical TAM signalling in macrophages and dendritic cells. In TME, stromal and tumour cells produce high concentrations of TAM ligands. This permits active TAM signalling in immune cells in TME and in tumour cells and renders them not sensitive to the decoy functions of sTAM.

Thus, in contrast with the reduced TAM signalling in autoimmune conditions, it must remain high in immune cells in TME. It is likely that production of TAM ligands by cancer cells or by tumour-educated macrophages exceeds the levels of sTAM in TME. This model was discussed in the context of hepatocellular carcinoma producing high levels of sAXL [127]. In this scenario, TAM signalling in cancer cells and in the immune cells in TME would not be inactivated by the shedding, and the production of sTAM would merely reflect the high expression of the receptors on the tumour cell surface and indicate overactivated MAPK or PKC signalling upstream of ADAM10/17 (Fig. 3). This model is in accord with enhanced levels of TAM ligands detected in biological fluids of cancer patients (Table 2) and reduced ligand levels associated with autoimmune diseases (Table 1). In tumours with high apoptotic indices, sTAM and TAM ligands can become sequestered on the surface of apoptotic cells, accumulating in TME and masking the extent of TAM proteolysis and the levels of secreted ligands. Further studies can investigate the correlation between the levels of circulating TAMs, their presence in TME and the degree of apoptosis.

## TAM processing and non-canonical pathways in tumour cells

Soluble receptors may acquire new capabilities and play a role in signalling in a manner different from the corresponding membranous receptors. For example, the soluble pro-renin receptor is a ligand for Frizzled 8. Pro-renin/Frizzled 8 interaction stimulates canonical WNT pathways and transcription of the aquaporin 2 gene to regulate urine concentration [97]. In some cases, soluble receptor/ligand complexes have enhanced affinity to a co-receptor, and these ternary complexes activate downstream pathways (reviewed in [130]). However, to our knowledge, no data supports that sTAM, alone or in a complex with their ligands, interact with cell-surface receptors.

**Fig. 4:**
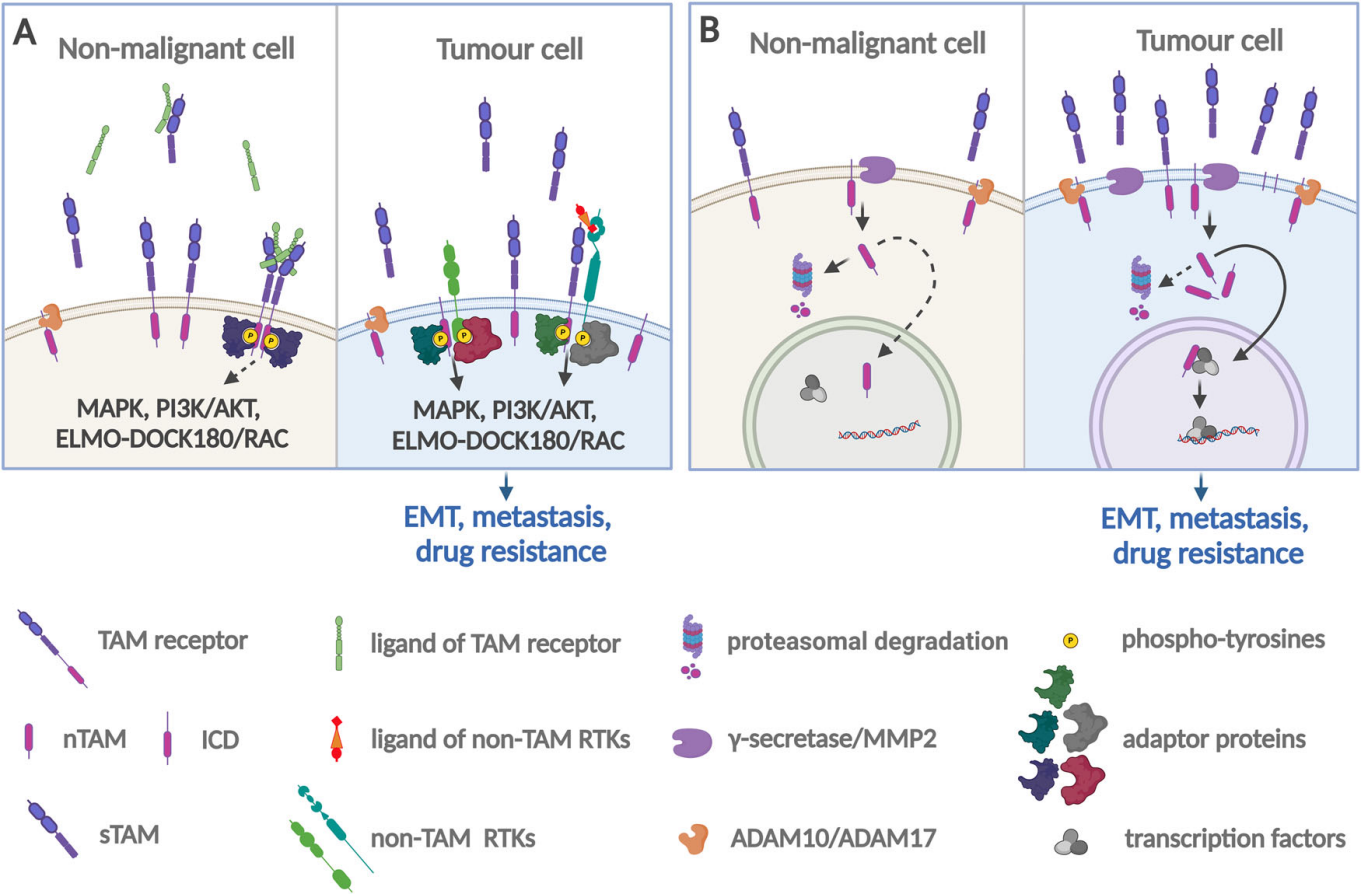
Switches from canonical to non-canonical pathways in cancer. **A** High expression of different RTKs in cancer cells determines a switch from ligand-dependent to ligand-independent signalling. TAM receptors may form heterodimers with unrelated RTKs and engage in ligand-dependent or -independent pathways. We propose that membrane-tethered intracellular domains of TAM receptors may heterodimerise with other RTKs and maintain signalling capacity. **B** Receptor shedding in cancer cells induces regulated intramembrane proteolysis and nuclear entry of soluble cytoplasmic TAM receptor fragments retaining kinase activity.

It is plausible to speculate that TAM receptors signal differently in non-malignant and cancer cells, where non-canonical pathways dominate. These non-canonical pathways are induced by the heterodimerisation of TAM receptors with different RTKs and are either ligand-independent or require interactions with ligands different from GAS6 or PROS1 (Fig. 4A). This type of signalling is predominantly relevant to AXL known to form heterodimers with TYRO3 [131, 132] and also with the unrelated RTKs, including EGFR and HER2, PDGFR, cMET, and VEGFR [87, 133–135]. In particular, AXL co-clustered with and transactivated EGFR, HER2, and cMET in cells of the mesenchymal subtype of ovarian cancer [87]. We also propose that following ED shedding, membrane-tethered intracellular TAM domains may preserve signalling functions independent of TAM ligands (Fig. 4A). This is reminiscent of truncated EGFR mutant, EGFRvIII, which stimulates downstream signalling in glioblastoma through dimerization with wt-EGFR [136, 137] or unrelated RTKs, such as cMET [136]. Likewise, the truncated version of HER2 receptor, p95-HER2, forms homo- or heterodimers with other EGFR family members in breast cancer [138]. Therefore, we propose that the availability of TAM-interacting RTKs defines the outcome of ED shedding. In cancer cells, where the expression levels of RTKs are high, ED shedding manifests a switch from the canonical to non-canonical GAS6- and PROS1-independent pathways via the dimerization of either full-size or truncated TAM receptors with other RTKs. In non-malignant cells with lower representations of RTKs, proteolysis of the receptors suppresses TAM signalling (Figs. 3, 4A).

We can envisage another scenario, whereby differences in the effects of TAM ED shedding between non-malignant and tumour cells are related to the generation of soluble ICD by RIP and their nuclear import (Fig. 4B). For all three TAM receptors, RIP is caused by γ-secretase (for AXL, MER, TYRO3) or MMP2 (for TYRO3) (see“sTAM and autoimmune diseases”). γ-secretase is a multisubunit complex that activates NOTCH and other molecular pathways implicated in cancer [139, 140]. Likewise, enhanced expression and activity of MMP2 have been reported in different cancer types, in association with poor survival in many cases [141, 142]. Because ED shedding is a prerequisite for RIP, the generation of sTAM leads to the generation of nTAM pools, specifically in tumour cells with active γ-secretase or MMP2 (Fig. 4B). In addition, emerging evidence has demonstrated that different components of the nuclear transport machinery are dysregulated in cancer [143]. Dysregulated transport across the nuclear membrane may be responsible for nTAM-mediated oncogenic signalling in cancer but not in non-malignant immune cells [144].

Although the protumourigenic role of TAM signalling is well established in most cancer types studied to date, it may provide unexpected tumour-suppressive effects in certain situations. Although TAM inhibition in several CRC cell lines reduced their tumourigenic features in vitro [145], the in vivo effect was different. Homozygous deletion of *Gas6* or double *Mer*^-/-^*Axl*^-/-^ knockout increased the amounts and sizes of intestinal polyps and shortened the survival of the mice in azoxymethane/dextran sulphate sodium-induced animal model of IBD and IBD-associated tumourigenesis [146, 147]. This tumour suppressive effect of TAM signalling is explained by the fact that chronic inflammation promotes tumourigenesis in many instances [148]. Therefore, the association between the high levels of sTAM and IBD-associated cancer can be explained by their decoy function leading to the formation of tumour-supporting inflammatory environment.

## Conclusion and clinical perspective

ED shedding inhibits TAM signalling in immune cells. Given the established role of TAM receptors in limiting immune responses, sTAM concentrations are expected to be elevated in the blood of patients with autoimmune and chronic inflammatory diseases. In cancer, the association between high levels of sTAM in biological fluids and disease aggressiveness may seem counterintuitive. Several reasons can explain this puzzling association. Firstly, high levels of TAM ligands in TME may make tumours insensitive to the decoy function of sTAM, and active ED shedding would indicate activation of oncogenic pathways upstream of sheddases in tumour cells. Secondly, non-canonical TAM signalling pathways may operate in tumour cells. As demonstrated in several studies, ED shedding is active in tumour cells in vitro. Although additional experimental proof is required, it is plausible to propose that the source of ED shedding in vivo is also tumour cells, which may lead to the activation of pathways driven by heterodimerisation of truncated TAM with other RTKs. Cleavage TAM receptors may also cause the formation of nTAM via RIP and activating protumourigenic programs, as shown for nTYRO3. Thirdly, one cannot exclude that inhibiting TAM signalling in immune cells by ED shedding may promote chronic inflammation, thereby facilitating the formation and progression of certain cancers. Further research using animal models and patients-derived xenografts will test these hypotheses and shed light on the physiological consequences of the proteolysis of TAM receptors in different cancer types.

More than twenty TAM receptor inhibitors are currently in various stages of development, from preclinical investigations to clinical trials [27, 28]. Most of them are type I or II small molecule inhibitors targeting the kinase activity of TAM receptors. Others represent anti-TAM aptamers or antibody-drug conjugates targeting cells expressing TAM receptors. A different strategy was used by Karolis et al., who applied unbiased mutagenesis to enhance the affinity of sAXL to GAS6. The engineered high affinity sAXL decoy receptor, Batiraxcept, displayed antitumor efficacy with low toxicity in preclinical models of advanced pancreatic and ovarian cancer [149, 150]. TAM receptors are often suppressed in autoimmunity, so their agonists may represent an approach to tackle these disorders. In support of this consideration, applying TAM ligands reduced the symptoms of collagen-induced arthritis in a mouse model [151]. Interestingly, the efficacy of ADAM10 inhibition was recently demonstrated in the same collagen-induced arthritis model [152]. However, this study did not address the effect of ADAM10 inhibition on TAM receptors.

Soluble forms of TAM receptors may be valuable as liquid biomarkers in clinics. It remains to be determined in clinical studies whether the presence of sTAM in biological fluids can help to stratify patients benefiting from TAM-targeted therapy. In support of this approach, treatment with a small molecule inhibitor, BGB324, reduced the production of sAXL by melanoma cells in vitro [126]. Further research is needed to establish if alterations in sTAM levels may inform about the response of patients with cancer or autoimmune conditions to different TAM antagonists or agonists.
